# Emotional intelligence and the dark triad: a latent profile analysis to investigate the Jekyll and Hyde of the emotionally intelligent manager

**DOI:** 10.3389/fpsyg.2024.1459997

**Published:** 2024-12-02

**Authors:** Christophe Haag, Clément Poirier, Lisa Bellinghausen

**Affiliations:** ^1^Emlyon, Lyon, France; ^2^Generation QE, Vannes, France; ^3^Laboratoire de Psychologie Appliquée et d’Ergonomie, Institut de Psychologie, Université Paris Descartes, Paris, France; ^4^Moodwork, Paris, France; ^5^Qualia Emotion Institute, Lyon, France

**Keywords:** emotional intelligence, manager ability, latent profile analysis, dark triad (DT), ability emotional intelligence

## Abstract

Managers sometimes have a bad reputation as they are often perceived as more manipulative than other employees. This study focuses on the Dark Triad (DT)—comprising psychopathy, Machiavellianism, and narcissism—and its connection with managers’ “Ability” Emotional Intelligence (AEI). The link between DT (measured through the Dirty Dozen) and managers’ AEI (measured through QEPro, an AEI performance test) was examined through a Latent Profile Analysis (LPA). We identified two AEI latent profiles within a heterogeneous population of 231 French managers. Our results show that managers with the FEP (Full Emotional Processing) profile are less Machiavellian (relative to the MEP -Minimal Emotion Processing- profile). Our results show that identifying AEI profiles may be a practical way to prevent toxic Management.

## 1 Introduction

### 1.1 The dark manager

*“36 percent of American workers have a boss whose leadership style could be described as “dysfunctional.” So, if you have long suspected that your boss is nuts, you might be right.” Time, February 3, 2015.*

People with power are often perceived as dark (Machiavelli, 2003; Rose et al., 2015; Sutton, 2007). Managers are no exception to this rule as they are often perceived as more manipulative than other employees (Baktash and Jirjahn, 2023; Sutton, 2010). In such a vein, some research has revealed a positive relationship between Machiavellianism and holding a managerial position (Baktash and Jirjahn, 2023; Rose et al., 2015). Further, research shows that managers with narcissistic and psychopathic tendencies tend to engage in bullying behaviors, contribute to depression (Tokarev et al., 2017) and hinder career advancement among subordinates (Volmer et al., 2017). The Machiavellianism, psychopathy and narcissism traits mentioned above belong to the so-called concept of the Dark Triad of personality (DT) (Furnham et al., 2013; Paulhus and Williams, 2002). These three traits have been identified in the literature as aversive, distinct and yet interconnected, each underpinned by an important emotional dimension (Paulhus and Williams, 2002). This emotional dimension is considered a “black box” that scholars have not fully illuminated to explain managers’ capacity to harm others (Palmer et al., 2020). In response, we explore the association between the managers’ levels of both dark traits and emotional intelligence.

#### 1.1.1 The psychopathic manager

Managers with a psychopathic personality tend to be characterized by a reduced reactivity to emotions (Patrick et al., 1994). Psychopaths exhibit weak connectivity between the ventromedial prefrontal cortex and the amygdala, crucial areas for emotions like guilt, remorse, and empathy (Patrick et al., 1994). While psychopaths may experience emotions, their “default” emotional configuration differs (Keysers and Gazzola, 2014).

Managers with a psychopathic personality are more likely to be malicious than average and derive a form of satisfaction from humiliating (Clarke, 2005) and harming others (O’Boyle et al., 2012). They are characterized by a certain harshness and callousness (Levenson et al., 1995). They exhibit primary selfishness, antisocial behavior, impulsivity, and an unstable lifestyle (Jones and Paulhus, 2014).

#### 1.1.2 The narcissistic manager

Managers with a narcissistic personality are inclined to display a lack of empathy, tend to be megalomaniacal, and selfish. They have a high opinion of themselves (self-overestimation) and are willing to do anything to maintain it (Morf and Rhodenwalt, 2001), not hesitating in the workplace to criticize others (even though they are themselves highly sensitive to criticism when it is aimed at them), exaggerate their accomplishments, and to take credit for the work and successes of others (Morf and Rhodenwalt, 2001).

#### 1.1.3 The Machiavellian manager

Managers with a Machiavellian personality tend to seek to manipulate emotionally and exploit others to serve their own interests, without regard for the emotional consequences of their actions on others or the moral dimension of their behaviors (Jakobwitz and Egan, 2006).

### 1.2 Emotional intelligence and the dark triad

*“In its extreme form, EQ is sheer Machiavellianism—the art of socially manipulating others in order to achieve one’s own selfish ends. When used in this way, other people become social tools to be used to push oneself forward even at considerable expense to them.” Psychology Today, August 15, 2014.*

Given the emotional dimension of the three dark triad components, an emerging academic discourse has debated the association between emotional intelligence (EI) and DT (Jauk et al., 2016). EI, first coined by Salovey and Mayer (1990), is defined as “the ability to monitor one’s own and others’ feelings and emotions, to discriminate among them and use this information to guide one’s thinking and actions.” It is the “Monitoring others’ feelings’ ability of EI that often raises questions about the dark side of this form of intelligence (Côté et al., 2011).

Researchers, though rare, found a positive link between emotional intelligence and specific Dark Triad traits, often focusing on EI’s subdimensions such as emotional regulation (Bianchi et al., 2020; Côté et al., 2011; Davis and Nichols, 2016). They suggest that individuals who control their emotions and are able to influence those of others may take advantage of this ability and use it to manipulate others as they wish, serving their own interests in certain situations (Côté et al., 2011; Davis and Nichols, 2016). Conversely, a vast majority of studies found a negative correlation between core components of emotional intelligence and the Dark Triad traits (Michels and Schulze, 2021), positing for example that psychopathic individuals, in particular, may be limited in empathy, considered by some researchers as a component of EI (Di Lorenzo et al., 2019; van Dongen, 2020).

This article contributes to this debate by investigating whether managers with high EI employ this form of intelligence to manipulate others, or if their EI serves as a protective factor against toxic management.

#### 1.2.1 Emotional intelligence as an ability

Two approaches to EI coexist today: (a) “Ability” EI (AEI) (Mayer et al., 2004; Haag et al., 2023) and (b) “Trait” EI (TEI) (Bar-On, 1997; Goleman, 1995; Petrides and Furnham, 2000, 2003). These two perspectives of EI have been found to weakly correlate with each other (Ferguson and Austin, 2010; Haag et al., 2023; Mayer et al., 2016) and the AEI approach has been considered more promising than the TEI (Haag et al., 2023; Mayer et al., 2016; Schlegel and Mortillaro, 2019). AEI is considered a form of intelligence among others whereas TEI is defined as a “personality trait” among others with key conceptual and psychometric limitations (Schlegel and Mortillaro, 2019).

Notably, Jauk et al. (2016) pointed out that the vast majority of studies investigating the association between EI and DT have relied on TEI. Few have employed an AEI approach, especially within the managerial context. Further, none of these studies has explored the link between the DT and AEI profiles using a Latent Profile Analysis (LPA) to identify which AEI profiles are the most/less associated with DT. This is in line with Cragun et al. (2020) who recently called on researchers to provide fresh insights concerning the Dark Triad in the management context.

This exploratory article aims to fill these gaps.

#### 1.2.2 AEI profiles based on the QEPro model

Almost 30 years after their founding article of AIE, Mayer et al. (2016) began refining the theoretical understanding of the AEI model and its measurement. In such a vein, Haag et al. (2023) proposed the QEPro model, an AEI extended model of Mayer and Salovey (1997) elaborated through theory-based item development and a scoring method as well as within the Situational Judgment Tests (SJT) framework to fit the management realm. According to the QEPro Model, the AEI of the manager is composed of the seven dimensions listed below:

- *Scanning Physiological Manifestations*: the ability for a manager to recognize emotions by analyzing the body sensations s/he experiences.
- *Interpreting Emotional Cues*: the ability for a manager to identify emotions through their cognitive manifestations, and other cues (e.g., vocal, postural and facial expressions).
- *Identifying Emotional Triggers*: the ability for a manager to identify what causes the emergence of emotions in self and in others.
- *Understanding Emotional Timelines*: the ability for a manager to evaluate accurately the intensity of emotions and to predict how emotions change (in terms of intensity) over time.
- *Anticipating Emotional Outcomes*: the ability for a manager to anticipate emotions’ consequences (positive or negative).
- *Selecting the Target Emotional State*: the ability for a manager to choose the suitable emotional state (called “target” emotional state) for a specific situation.
- *Emotion Regulation*: the ability for a manager to apply the accurate emotion regulation strategy to reach the target emotional state.

According to Fino et al. (2023), using a person-centered approach through LPA (Latent Profile Analysis) can help to clarify the interplay between Dark traits of personality and EI dimensions. Indeed, LPA—which is specifically designed to account for the presence of subpopulations characterized by different parameters (Meyer and Morin, 2016; Morin, 2016) - offers a unique way to explore how many and which AEI profiles may emerge. In the present research, we therefore use LPA to identify such emerging AEI profiles (based on the QEPro’s seven dimensions described above) among a population of managers and then explore their relationships with dark traits (psychopathy, Machiavellianism, and narcissism).

#### 1.2.3 Hypotheses

In their meta-analysis, Gómez-Leal et al. (2018) found a negative relationship between AEI and psychopathy in the majority of the studies reviewed. In another meta-analysis (including studies using both AEI and TEI approaches), Tsirimokou et al. (2021) found that high AEI levels are associated with low Machiavellianism levels. Additionally, Nguyen et al. (2022) found through their meta-analysis that narcissism was negatively associated with AEI. Finally, some researchers postulate that emotionally intelligent managers tend to contaminate their subordinates with beneficial emotions rather than toxic ones (Haag and Getz, 2016; Haag and Wolff, 2024).

Based on the above-mentioned literature, we formulate three hypotheses:

*H1*: The emotionally intelligent manager, characterized by an AEI profile that outperforms the other profiles on the QEPro’s seven dimensions, is less psychopathic (relative to the other profiles).

*H2*: The emotionally intelligent manager, characterized by an AEI profile that outperforms the other profiles on the QEPro’s seven dimensions, is less narcissistic (relative to the other profiles).

*H3*: The emotionally intelligent manager, characterized by an AEI profile that outperforms the other profiles on the QEPro’s seven dimensions, is less Machiavellian (relative to the other profiles).

## 2 Method

### 2.1 Population

The study included 231 French managers (115 men, 116 women) with an average age of 43 years (*SD* = 7.9). Participants were distributed across various company sizes, with 15% in small enterprises, 26% in medium-sized enterprises, 30% in intermediate-sized enterprises, and 29% in large enterprises. The managers had varying levels of experience, ranging from less than 6 years to over 20 years. They were recruited using social networks (mainly through Linkedin).

### 2.2 Measures

#### 2.2.1 Participants completed AEI and DT questionnaires online (via Qualtrics)

##### 2.2.1.1 Managers’ AEI measure

EI can be measured in two different ways, depending on the model used (AEI or TEI). The way of assessing TEI involves self-reported measures (Likert-style responses to items) that face key limitations such as *social desirability* (Day and Carroll, 2004; Matthews et al., 2004) and participants’ difficulty in having sufficient objective perspective on oneself (Brackett et al., 2006; Kruger and Dunning, 1999; Sheldon et al., 2014). Moreover, TEI measures “violate the first law of intelligence” (Schlegel and Mortillaro, 2019, p. 560) because of their significant correlations with personality measures (Alegre et al., 2019; Matthews et al., 2004) and their lack of correlation with cognitive intelligence (Furnham and Petrides, 2003). Despite these key issues, researchers often use TEI questionnaires because of their ease of administration (short questionnaires that are less cognitively demanding and time-consuming than AEI performance tests).

On the other hand, AEI is assessed through performance tests (Haag et al., 2023; Haag et al., 2024) that “measure individual performance in solving emotional problems and performing emotional tasks” (Haag et al., 2023, p. 4082) making these tests more “objective” than TEI self-report questionnaires (Zeidner et al., 2008). Among these AEI performance tests is the QEPro, a questionnaire elaborated within the Situational Judgment Tests (SJT) framework that is specially designed for managers and related to the QEPro model (Haag et al., 2023).

The QEPro performance test consists of 35 items each with correct and incorrect response options. For example, the “Interpreting Emotional Cues” dimension is measured through five items. Each item describes an emotion based on three or four core emotional cues. The test-taker is asked to select which emotion (among the options proposed) is described in the item.

E.g. “A pleasant warmth invades my face and my chest, and my voice is characterized by great loudness, high pitch, and fast speed. I am upright and I want to celebrate this feeling with those around me.”

(a) Pride; (b) Joy; (c) Satisfaction; (d) Surprise; (e) Hope; (f) Awe.

QEPro has good psychometric qualities such as an appropriate level of difficulty (the minimum of the items’ difficulty level is set at 0.20; item discrimination ranges from 0.20 < D < 0.80) and a clear factorial structure (investigated both at the item level and the dimension level; all fit indexes are acceptable to excellent). The questionnaire also correlates in meaningful and theoretically congruent ways with different Affect measures (e.g., Alexithymia, TAS-20: *r* = −0.13, *p* < 0.01 and Empathy, BES-A: *r* = 0.18, *p* < 0.01). As expected, no significant correlations were found between QEPro and TEI self-report measures (e.g., TEIQue: *r* = 0.03, n.s.) and the Big Five factors of personality (BFI: ranging from −0.03 to 0.06, n.s.). It is also notable that QEPro is positively but moderately correlated with general intelligence, as measured by the RAVEN’S™ Advanced Progressive Matrices (APM-SF: *r* = 0.28, *p* < 0.01). Therefore, we can consider that QEPro measures a form of intelligence that is distinct from general intelligence (for more details see Haag et al., 2023). We found good internal consistency for QEPro in our sample of 231 managers (*α* = 0.85).

### 2.3 DT measure

The Dirty Dozen (DTDD-FC; French-Canadian adaptation by Savard et al., 2017) is a short self-reported questionnaire that assesses the Dark Triad traits, namely psychopathy (4 items; *α* = 0.72), Machiavellianism (4 items; *α* = 0.82) and narcissism (4 items; *α* = 0.84). It consists of 12 items, each rated on a 5-point Likert-type scale.

#### 2.3.1 Analysis

First, we ran a Latent Profile Analysis (LPA) to identify AEI latent profiles. LPA is designed to determine sub-groups within an extent sample, called profiles. LPA was run using R software with the Mclust package (Scrucca et al., 2016) to investigate one to eight profiles. The optimal number of profiles was selected based on statistical criterions (AIC, BIC, CAIC, ABIC), substantive meaningfulness and theoretical conformity (Gillet et al., 2019).

Second, we examined the differences between the AEI profiles regarding their respective association with the Dirty Dozen dimensions (psychopathy, Machiavellianism, and narcissism). We used several student’s *t*-tests with R software for this step, one for each dimension.

## 3 Results

### 3.1 Latent profile analysis (LPA)

The statistical criteria are indicated in Table 1 for each profile (between 1 to 8 solutions). The 2-profile to the 5-profile solutions were examined more closely. This examination demonstrated that the 2-profile solution was the most proper solution regarding its statistical and theoretical conformity. Indeed, the 2-profile solution presented the lowest BIC and an acceptable entropy.

**Table 1:** Parameters of latent profiles analyses.

Classes	AIC	BIC	CAIC	Entropy	BLRT
1	5317.6	5365.5	5379.5	1	
2	5212.7	5288.4	5310.4	0.80	120.5**
3	5214.6	5317.8	5347.9	0.73	14.0
4	5192.6	5323.4	5361.4	0.71	38.0**
5	5180.6	5338.9	5384.9	0.74	28.0**
6	5184.1	5369.9	5423.9	0.74	12.5
7	5180.1	5393.5	5455.5	0.74	19.9
8	5180.6	5491.6	5491.6	0.75	15.4

**p < 0.01.

**Figure 1:**
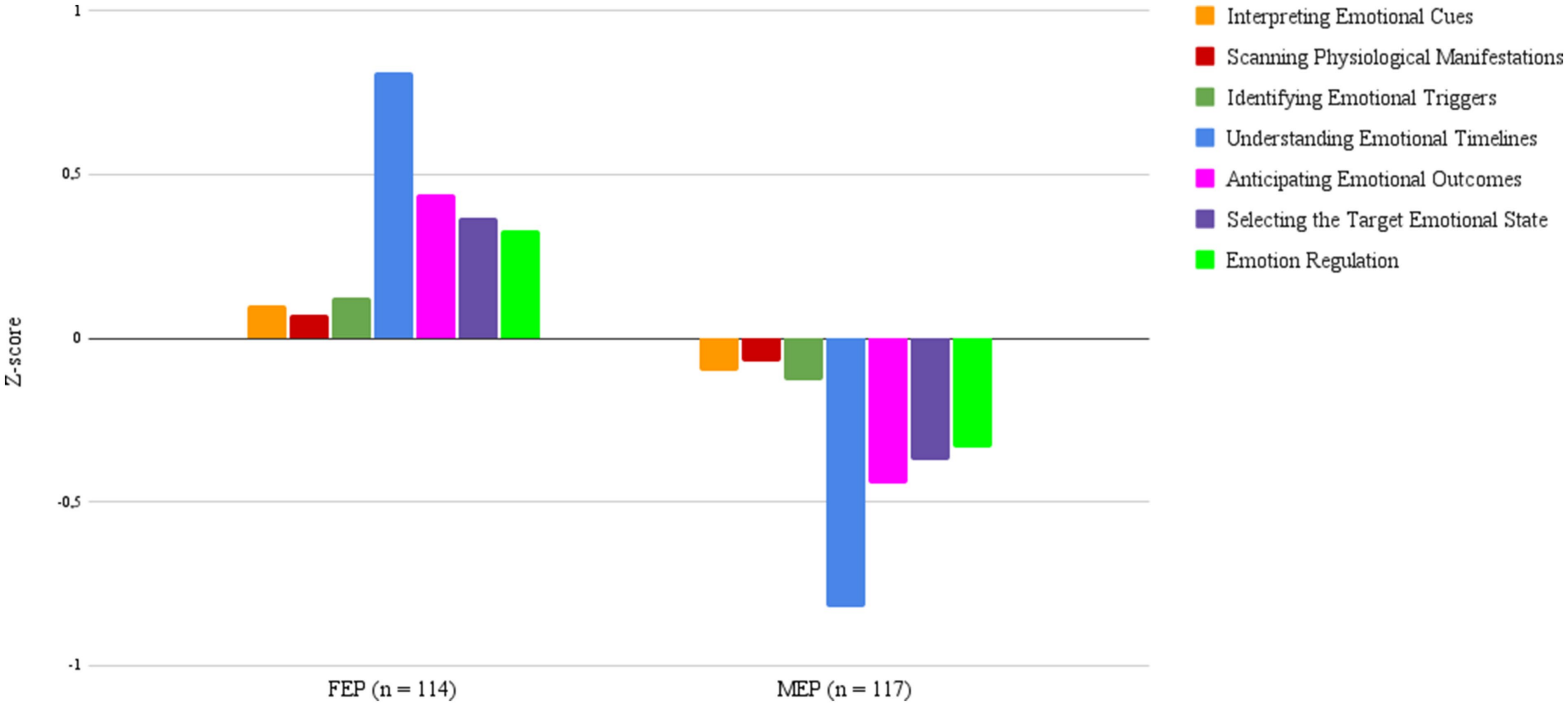
Comparison two profile solution based on QEPro’s seven sub-dimensions (FEP: Full Emotion Processing profile; MEP: Minimal Emotion Processing profile).

Two AEI profiles for managers emerged (Figure 1), namely FEP (“Full Emotional Processing”) and MEP (“Minimal Emotional Processing”). The FEP profile (*n* = 114) significantly outperformed the MEP profile (*n* = 117) on five out of the QEPro’s seven dimensions (Table 2). Thus, FEP displays the most complete processing of emotion as managers with this profile appear to be more capable of identifying, understanding and managing emotions.

**Table 2:** Means and student’s *t*-test results for QEPro dimensions by AEI profiles.

QEPro dimensions	FEP	MEP	*t*	Cohens’ d
Interpreting Emotional Cues	0.1	−0.09	1.51	0.19
Scanning Physiological Manifestations	0.02	−0.02	0.34	0.05
Identifying Emotional Triggers	0.16	−0.15	2.39*	0.32
Understanding Emotional Timelines	0.85	−0.83	23.7***	0.91
Anticipating Emotional Outcomes	0.46	−0.45	7.7***	0.83
Selecting the Target Emotional State	0.39	−0.38	6.26***	0.82
Emotion Regulation	0.34	−0.33	5.41***	0.71

* *p* < 0.05, *** *p* < 0.001, FEP: Full Emotion Processing profile, MEP: Minimal Emotion Processing.

These two profiles were similar regarding the gender distribution (χ^2^ (1, 231) = 3.6, *p* = 0.06) and the age distribution (t (1, 231) = −0.81, *p* = 0.42).

#### 3.1.1 AEI profiles and Machiavellianism

We found that the FEP (relative to the MEP profile) had the lowest Machiavellianism score (see Table 3), which supports H3. To summarize, FEP managers (i.e., managers who score high on each of the seven dimensions of EI), are less Machiavellian than the MEP profile. This suggests that FEP managers tend to put their abilities to accurately identify, understand and regulate emotions at the service of self as well as others, consequently impacting positively their work environment. They are more prone to constructive (rather than destructive) leadership behaviors.

**Table 3:** Means and student’s *t*-test results for Dirty Dozen dimensions by AEI profiles.

Dirty Dozen dimensions	FEP	MEP	*t*	Cohens’ d
Psychopathy	11.0	11.8	−1.1	0.14
Machiavellianism	11.8	13.8	−2.4*	0.30
Narcissism	18.2	18.1	0.18	0.03

* *p* < 0.05, FEP: Full Emotional Processing profile, MEP: Minimal Emotional Processing.

No significant associations were found between the AEI profiles and the other components of DT (Psychopathy and Narcissism) to support H1 and H2.

## 4 Discussion

The aim of the present study was to explore the associations between AEI profiles and DT traits using LPA in a population of managers who are often perceived as dark individuals. We found two AEI profiles for managers, namely FEP and MEP.

Our results did not support H1 and H2. The non-support for H1 is consistent with two studies that found no relationship between AEI and psychopathy (Curci et al., 2017; Kahn et al., 2016). And the non-support for H2 is consistent with studies that revealed that EI was unrelated to narcissism (Czarna et al., 2016; Miao et al., 2019).

Our results support H3 by showing that the FEP profile is negatively and significantly associated with Machiavellianism. This is consistent with the meta-analysis of Tsirimokou et al. (2021) showing that high AEI levels are associated with low Machiavellianism levels.

However, our finding contrasts with former studies suggesting that AEI could play a significant role in effective manipulation (Côté et al., 2011; Kilduff et al., 2010; Schlegel, 2020). Researchers who highlighted the dark side of AEI explained that individuals who understand how emotions are processed and how to regulate them in one’s and others tend to take advantage of this capacity to manipulate others as they wish, preferring to serve their own interests in certain situations (Côté et al., 2011; Davis and Nichols, 2016). Moreover, as a crucial component of AEI is the capacity to accurately recognize others’ emotions (Salovey and Mayer, 1990), one could argue that such an ability allows manipulators to recognize the needs and interests of their targets in everyday situations (Konrath et al., 2014). Consequently, Bianchi et al. (2020) argued that AEI could increase a manipulator’s ability to discern what their targets value or fear, enjoy or hate, assess their strengths and weaknesses, or evoke emotions such as guilt and obligation, which can make individuals more compliant.

Our results are not in line with the above-mentioned interpretations. However, FEP managers master all the EI abilities -described above- supposed to make them good manipulators. A first explanation is that Machiavellianism has been linked to emotional dysfunctions such as alexithymia -difficulty in identifying, differentiating, and expressing emotions, both one’s own and others’- and anhedonia -inability to experience positive affect- (Al Aïn et al., 2013). Yet, both -Alexithymia and Anhedonia- are negatively associated with EI (Abdollahi et al., 2020; Di Lorenzo et al., 2019; Haag et al., 2023). Additionally, research has established positive associations between Machiavellianism and a lack of empathy (Blötner et al., 2021), the latter being correlated with a lack of AEI (Haag et al., 2023). Empathetic individuals, if they harm others, would in turn feel the pain caused to others (the boot is on the other foot now) which could deteriorate their mental health and the quality of their relationships with others over time. However, previous research showed that AEI is significantly and positively associated with better mental health (Zeidner and Matthews, 2016) and good relationships (Lopes et al., 2003).

To conclude, our result showed that AEI is not “sheer Machiavellianism” contrary to what was stated in the *Psychology Today*’s quote. While early empirical studies support this relationship among adolescents (Zhang et al., 2015) and college students (Jauk et al., 2016), our study extended these results to a population of managers, often perceived as dark, using an AEI latent profile approach.

### 4.1 Limitations and future research

Even though our sample is composed of a respectable number of participants (*N* = 231) who are managers not easy to convince to fill out questionnaires due to their busy schedules, we intend to increase this number in future research to strengthen the generalizability of our results. We also intend to increase the sample size of underrepresented organizations (e.g., small enterprises) in future research to generalize the findings to different cultural or organizational contexts.

In our sample, no extreme cases of DT (individuals with very high scores on each DT trait) were identified. We further intend to examine the relationship between EI and DT by using the extreme groups approach (EGA), resulting in an extreme groups design characterized by the presence of individuals with extreme scores on DT. This will allow us to observe the associations between very high scores on DT dimensions and the scores on one or several EI dimensions. We can assume that someone with extreme DT scores could potentially master one or several dimensions of EI but rarely presents a full emotional processing (FEP) profile (Côté et al., 2011). Therefore, it’s important to carry out research that includes these extreme cases. Further studies could be conducted with populations already identified as manipulative, Machiavellian or psychopathic. These results could deepen our understanding of the relationships observed in this study and provide a better comprehension of the different emotional characteristics of these kinds of individuals and their destructive power within organizations.

Past research has already focused on the link between EI and the Dark Triad. Our work extends this work as few studies focused on AEI (rather than TEI) when regarding DT, moreover, by adopting a person-centered approach. While AEI is not theoretically linked to personality traits (Mayer et al., 2016), researchers study its relationship with the Dark Triad to explore how emotional abilities can intersect with certain behaviors associated with these traits. Studies of this kind should be pursued in order to better understand how individuals with high AEI respond to or manage the interpersonal challenges posed by Dark Triad traits.

Although the Latent Profile Analysis (LPA) is valuable, we intend in future research to incorporate qualitative data and/or case studies to improve the ecological validity of our study and refine our findings.

The Dark Triad has been recently extended with the addition of a fourth dark trait, namely Sadism, completing the so-called Dark Tetrad (Bonfá-Araujo et al., 2022). We invite researchers to investigate the relationship of this trait (and the whole concept of Dark tetrad) with EI profiles.

Further research could also focus on the relationship between emotional intelligence and The light triad (devised in contrast to DT) which consists of Kantianism, humanism, and faith in humanity (Kaufman et al., 2019).

### 4.2 Implications for managers

Researchers showed that it is possible to increase managers’ AEI through training (Gilar-Corbi et al., 2019) - such a process was labeled “emotional plasticity” (Kotsou et al., 2011). The development of AEI training programs based on QEPro and its underlying AEI model could help managers to move closer to the FEP profile, helping them to fully process emotions, and thus reduce their tendency to manipulate emotionally and exploit others to serve their own interests. Developing full emotional processing of others’ feelings will strengthen the managers ability to identify, understand and thus take into account the emotional consequences of their actions on others in order to behave ethically (Jakobwitz and Egan, 2006). This is in line with the literature showing that emotionally intelligent managers tend to adopt more ethical behaviors, mainly by avoiding harming others (Angelidis and Ibrahim, 2011; Hopkins and Deepa, 2018). If managers increase their AEI (e.g., through training), they should consequently increase their level of authenticity as research showed that high AEI levels are significantly and positively related to high levels of authentic leadership (Miao et al., 2019).

We also invite organizations to embrace emotional intelligence as a criterion for internal promotion and to integrate it into HR processes in order to promote positive respectful leadership behaviors. In such a vein, recruiters should select candidates for the position of managers based on their AEI profiles in order to prevent destructive management behaviors within organizations. Recruiters could administer QEPro tests or other valid AEI performance-based measures to objectively examine candidates’ ability to identify, to understand and to regulate emotions in themselves and others, thus reducing the risk of letting the wolf into the fold.

## Data Availability

The datasets presented in this study can be found in online repositories. The names of the repository/repositories and accession number(s) can be found at: https://osf.io/yrfb5/?view_only=0bd0ff11d5434315995ccf5f8209bb3d.
